# Retrospective observational study of the effects of residual neuromuscular blockade and sugammadex on motor-evoked potential monitoring during spine surgery in Japan

**DOI:** 10.1097/MD.0000000000030841

**Published:** 2022-09-30

**Authors:** Hironobu Hayashi, Miki Yamada, Kotoba Okuyama, Tsunenori Takatani, Hideki Shigematsu, Yasuhito Tanaka, Masahiko Kawaguchi

**Affiliations:** a Department of Anesthesiology, Nara Medical University, Nara, Japan; b MSD K.K., Tokyo, Japan; c Division of Central Operation, Nara Medical University Hospital, Nara, Japan; d Department of Orthopedic Surgery, Nara Medical University, Nara, Japan.

**Keywords:** intubation, motor-evoked potential, neuromuscular blockade, rocuronium, sugammadex

## Abstract

Given neuromuscular blockade (NMB) can affect the amplitude and detection success rate of motor-evoked potentials (MEP), sugammadex may be administered intraoperatively. We evaluated the factors affecting the degree of residual NMB (i.e., the train-of-four [TOF] ratio) and the relationship between TOF ratio and MEP detection success rate in Japanese patients undergoing spine surgery. This single-center retrospective observational study included adults who underwent spine surgery under propofol/remifentanil anesthesia, received rocuronium for intubation, and underwent myogenic MEP monitoring after transcranial stimulation. TOF ratios were assessed using electromyography. Sugammadex was administered after finishing the MEP setting and the TOF ratio was ≤0.7. To identify factors affecting the TOF ratio, TOF ratio and MEP detection success rate were simultaneously measured after finishing the MEP setting; to compare the time from intubation to the start of MEP monitoring after NMB recovery between sugammadex and spontaneous recovery groups, multivariable analyses were performed. Of 373 cases analyzed, sugammadex was administered to 221 (59.2%) cases. Age, blood pressure, hepatic impairment, and rocuronium dose were the main factors affecting the TOF ratio. Patients with higher TOF ratios (≥0.75) had higher MEP detection success rates. The time from intubation to the start of MEP monitoring after NMB recovery was significantly shorter in patients administered sugammadex versus patients without sugammadex (*P* < .0001). The MEP detection success rate was higher in patients with a TOF ratio of ≥0.75. Sugammadex shortened the time from intubation to the start of MEP monitoring after NMB recovery.

## 1. Introduction

Patients undergoing neurosurgery, such as spine surgery and craniotomy, and those undergoing aortic surgery are at high risk of suffering a movement disorder due to surgery-related injuries.^[1]^ Thus, motor-evoked potential (MEP) monitoring is recommended during surgery to prevent postoperative motor dysfunction.^[2–4]^ Furthermore, patients undergoing general anesthesia require endotracheal intubation; to safely perform intubation, these patients are administered a muscle relaxant, such as rocuronium, which considerably suppresses MEP.^[5–7]^ Thus, for surgeries in which MEP monitoring is planned, muscle relaxants should be used cautiously.

Sugammadex, a selective relaxant binding agent,^[8]^ reverses moderate and deep vecuronium- and rocuronium-induced neuromuscular blockade (NMB).^[5,9]^ Sugammadex was approved in Japan in 2010. Since then, its postoperative use to rapidly reverse moderate and deep muscle relaxation has become widespread, particularly as rocuronium is the only muscle relaxant currently used in Japan. Sugammadex is expected to shorten the recovery time from muscle relaxation.^[8]^ Recently, a study in Chinese patients undergoing spine surgery showed that sugammadex reversed rocuronium-induced NMB, and MEP amplitude was enhanced 5 minutes after dural opening.^[10]^

So far, no real-world studies on Japanese populations have assessed the effect of NMB on MEP amplitude and the extent to which sugammadex shortens the time of recovery from NMB intraoperatively. We hypothesized that patients with a lower train-of-four (TOF) ratio would have a lower MEP detection success rate at the first MEP measurement. Thus, this retrospective observational study aimed to characterize patient-related factors that affect the residual NMB quantified by TOF ratio and to evaluate the relationship between the degree of residual NMB and MEP detection success rate in Japanese patients undergoing spine surgery. We also evaluated the effect of intraoperative administration of sugammadex on MEP amplitude and other parameters including the time from intubation to start of MEP monitoring after NMB recovery, operation time, and operating room stay time.

## 2. Methods

### 2.1. Ethical approval and research registration

The study was approved by the Independent Ethics Committee of Nara Medical University (approval number 2087) on December 17, 2018, and written informed consent was retrospectively obtained from all subjects. This study was registered at the University hospital Medical Information Network Clinical Trials Registry (identification number: UMIN000035799; principal investigator: Masahiko Kawaguchi; date of registration: March 1, 2019). Clinical research registration occurred prior to the start of this study.

### 2.2. Study design

This was a retrospective secondary data collection study conducted using medical record data from Nara Medical University. Patients were grouped according to whether they were administered sugammadex or not (hereafter referred to as the spontaneous recovery group). Given the retrospective and observational nature of the study, patient characteristics, their medical conditions, and anesthesia data were obtained from medical records, anesthesia charts, and MEP monitoring devices, collected in the context of routine clinical care between April 2013 and December 2018 (i.e., the data collection period). This data collection period was selected based on the expectation that proficiency for MEP monitoring during spine surgery would have been acquired by 2013.

Patient data were obtained from medical and anesthesia charts, including data on patient background characteristics such as sex, age, weight, height, and body mass index (BMI), as well as other clinical characteristics. American Society of Anesthesiologists classifications, surgical procedures, lesion site, comorbidities, presence or absence of preoperative motor paralysis (defined as a manual motor testing grading of ≤3), medications used, type and depth of anesthesia (bispectral index values [BIS]) (deep [<40], appropriate [≥40 to ≤60], or light anesthesia [≥61]) or patient state index (PSI) (deep [<25], appropriate [≥25 to ≤50], or light anesthesia [≥51]) for quantifying the depth of anesthesia), core body temperature (˚C) at the first MEP measurement after finishing the MEP setting, intraoperative position (supine or prone), mean blood pressure, and heart rate were also obtained from medical and anesthesia charts. Data from laboratory tests (e.g., creatinine, estimated glomerular filtration rate [eGFR], aspartate aminotransferase, alanine aminotransferase, fasting plasma glucose, glycosylated hemoglobin) were also collected. Renal impairment was defined as the presence of abnormal eGFR or creatinine clearance values. At Nara Medical University, the normal eGFR range is 60 to 120 mL·min^ − 1^·1.73 m^ − 2^; creatinine, 0.65 to 1.07 mg/dL; and creatinine clearance, 55.56 to 138.89 mL/min. Hepatic impairment was defined based on the Child-Pugh classification groups A (mild), B (moderate), or C (severe).

### 2.3. Measurements and protocols

As the study was observational, anesthesiology protocols were administered at the discretion of each anesthesiologist. All patients were maintained under total venous anesthesia with propofol and remifentanil. Rocuronium was administered before intubation, and if needed, additional doses were administered in response to body movements. Muscle relaxants, including succinylcholine, are not routinely used in Japan, and most facilities use rocuronium followed by sugammadex rather than neostigmine. Baseline for the sugammadex group was defined as the first MEP recording just after finishing the MEP setting and immediately before sugammadex administration. Sugammadex was administered after finishing the MEP setting and the TOF ratio was ≤0.7. MEP amplitude was measured in the left and right abductor pollicis brevis, tibialis anterior, soleus, and abductor hallucis, and was defined as the potential evoked (≥50 mV) by transcranial electrical motor stimulation and depolarization of pyramidal axons. The start of MEP monitoring after NMB recovery (generally 15 minutes after sugammadex administration) began when the patient recovered from muscle relaxation as measured using electromyography; their status was then changed to operable once a TOF rate >0.7 was confirmed, after which surgery was performed. For patients administered sugammadex, the following variables were measured at baseline and during the surgery (i.e., 5, 10, 15, 30, 60, and 120 minutes after sugammadex administration): TOF ratio, BIS or PSI, core body temperature, mean blood pressure, heart rate, MEP measurement muscles (in the left and right abductor pollicis brevis, tibialis anterior, soleus, and abductor hallucis), MEP amplitude at each muscle, and MEP stimulation intensity. The median nerve stimulation recording of the abductor pollicis brevis or the tibial nerve stimulation recording of the abductor hallucis was used for TOF measurement. TOF of the abductor pollicis brevis took precedence if both TOF measurements were taken. The TOF ratio was defined as the fourth response to the first response of a single stimulus given at 4 consecutive 2-Hz cycles (Neuromaster MEE-1232, Nihon Kohden, Tokyo, Japan). The procedure flow is shown in Supplemental Digital Content 1, http://links.lww.com/MD/H439. All surgeries were performed by a limited number of spine specialists from Nara Medical University who followed the same surgical protocol and were guided by one supervisor who was responsible for the orthopedic spine group.

### 2.4. Patients

Eligible patients were aged between 20 and 85 years, had undergone spine surgery (e.g., corrective fixation of scoliosis, spinal tumor removal, laminoplasty, posterior fusion, or fenestration) with myogenic MEP monitoring under total intravenous anesthesia at Nara Medical University within the data collection period, had received rocuronium prior to endotracheal intubation, had TOF measured by electromyography at the same time as MEP monitoring, and had provided informed consent to participate in this study. Patients were excluded if they were receiving anti-epileptic drugs or oral steroids at the time of spine surgery, as these drugs are known to affect neuromuscular function.

### 2.5. Primary outcomes

The first primary objective was to determine the effect different patient background factors had on NMB by evaluating the TOF ratio after finishing the MEP setting (i.e., at baseline). In addition, the second primary objective was to investigate the relationship between the TOF ratio category at baseline and MEP amplitude, which was evaluated using the MEP detection success rate after finishing the MEP setting (i.e., at baseline). Successful MEP detection was defined as successful detection at the left and right abductor pollicis brevis and abductor hallucis muscles, as well as detection at 2 or more left and right tibialis anterior and soleus muscles.

### 2.6. Exploratory outcomes

Exploratory outcomes included evaluating the degree of NMB recovery by sugammadex dose on the amplification of MEP amplitude using the measured MEP amplitude as an index. Other exploratory parameters were time from intubation to start of MEP monitoring after NMB recovery (defined as the time at which the MEP monitoring was performed after sugammadex administration, recovery from muscle relaxation had occurred, and the patient was considered to be in an operable status just before surgery), operation time, and operating room stay time (defined as the duration of time the patient remained in the operating room). Finally, variables that were measured at baseline, just before surgery, and after surgery were compared to validate sugammadex’s effectiveness versus patients in the spontaneous recovery group. In this analysis, baseline generally corresponded to the first MEP measurement after finishing the MEP setting in the spontaneous recovery group.

### 2.7. Methods for minimizing bias

As many factors may impact MEP monitoring, statistical testing and estimation were performed while adjusting for these effects by including these factors in the multivariable model. To minimize bias, the operating team consisted of spine specialists, and the method and procedure of MEP were consistent for all cases.

### 2.8. Statistical analysis

When assessing feasibility, we observed that 300 spine surgeries were conducted at our center between October 2016 and March 2018. Assuming the number of procedures per year remained constant, it was estimated that approximately 1000 spine surgeries would be conducted in 5 years. Of those, approximately 30% to 50% of patients would retrospectively provide informed consent to participate in this study, and 75% to 85% of patients would meet the eligibility criteria. Thus, the estimated and prespecified sample size ranged from 225 to 425 patients. Candidate variables for multivariable linear regression analysis for the first primary objective also included 16 variables. Multivariable linear regression analysis requires a total of 10 cases per variable, thus a minimum of 160 cases were required when all variables were included in the final model.^[9]^

Frequency tables (count and percentage of patients) were generated for categorical variables, and summary statistics (number [N], mean, standard deviation [SD], minimum, median, and maximum) were calculated for continuous variables.

For the first primary objective (primary analysis of patient background factors that contribute to higher values of TOF ratio), a multivariable linear regression model was performed with TOF ratio as the response variable and patient background characteristics (i.e., age, sex, BMI, mean blood pressure, heart rate, renal impairment, hepatic impairment, diabetes mellitus, preoperative motor palsy, rocuronium dose, propofol dose, remifentanil dose, BIS/PSI category (deep, appropriate, and light anesthesia), core body temperature, and time from rocuronium administration to TOF measurement) as explanatory variables. For the second primary objective (secondary analysis of the relationship between TOF ratio and MEP success rate after finishing the MEP setting), a multivariable logistic regression model was performed with MEP success rate after finishing the MEP setting as the response variable, and the TOF ratio category (<0.25; ≥0.25 to <0.5; ≥0.5 to <0.75; ≥0.75 to <0.9; and ≥0.9) and BIS/PSI category as explanatory variables. The effect of variables included in the primary analysis could reflect on the TOF ratio; therefore, only those that could independently affect the MEP success rate were included in the secondary analysis. Correlation coefficients between the TOF ratio (continuous) and MEP amplitude (actual) after finishing the MEP setting (i.e., before sugammadex administration) were calculated.

For the first and second primary analyses, the TOF ratio and MEP success rate after finishing the MEP setting (i.e., before sugammadex administration) were used to determine what factors impact residual NMB assessed by the TOF ratio (continuous variable), and the relationship between TOF ratio category and MEP success rate, respectively. Thus, these 2 analyses were cross-sectional in nature at baseline (i.e., before sugammadex administration) and combined data from both the sugammadex and spontaneous recovery groups after finishing the MEP setting. A post hoc analysis using different TOF ratio categories (<0.25; ≥0.25 to <0.5; ≥0.5 to <0.75; ≥0.75 to <0.9; and ≥0.9) was also performed using the MEP detection success rate after finishing the MEP setting.

For the analysis of exploratory variables, the MEP amplitude and the amplification rate of MEP amplitude (left-abductor pollicis brevis), summary statistics at each time point during surgery were calculated separately in the sugammadex and spontaneous recovery groups. For the analysis of other exploratory variables (time-related outcomes), a multivariable linear regression model was performed with the time-related variable as the response variable and patient background characteristics as explanatory variables. In these analyses, treatment group (sugammadex or spontaneous recovery) was added to the model as an explanatory variable to compare the 2 groups. Because most patients in the sugammadex group did not have the exact time for the start of MEP monitoring after NMB recovery, the time was conservatively set to 15 minutes after sugammadex administration for analysis, as clinical trial data show that sugammadex can affect recovery within 1 to 2 minutes.^[11–14]^

Prior to the regression analyses, correlation coefficients were used to examine potential collinearity between explanatory variables. The variable with the largest *P* value >.5 was then removed in order from the model by stepwise (backward) procedures. The final model was determined when there was no variable with a *P* value >.5 in the model to avoid the possibility of a false negative in the variable selection procedure.

As this study was not confirmatory, adjustments for multiplicity were not performed. All statistical analyses were performed using SAS version 9.4 (SAS Institute Inc., Cary, NC).

## 3. Results

### 3.1. Patient disposition and characteristics

**Figure 1.**
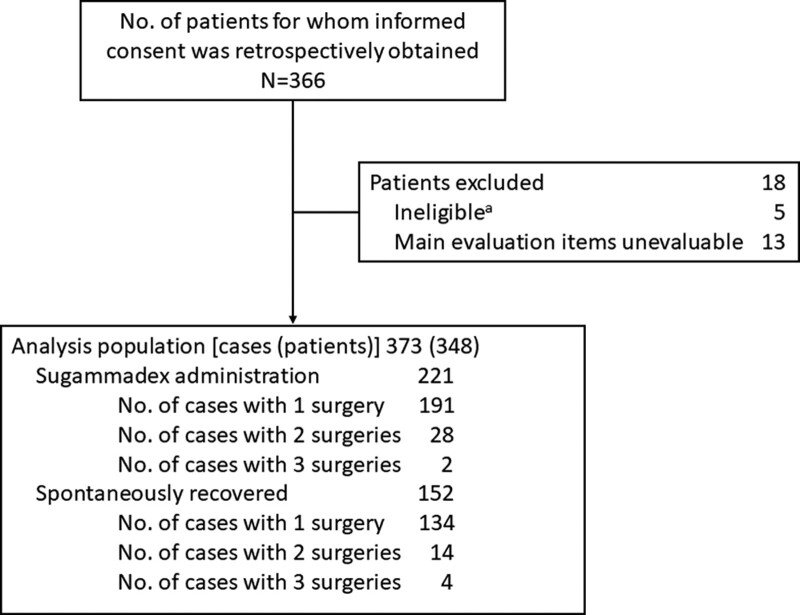
Patient disposition. ^a^Eligible patients were aged between 20 and 85 years, had undergone spine surgery with myogenic MEP monitoring under total intravenous anesthesia at Nara Medical University within the data collection period, had received rocuronium prior to endotracheal intubation, had TOF measured by EMG at the same time as MEP monitoring, and had provided informed consent to participate in this study. Patients were excluded if they were receiving anti-epileptic drugs or oral steroids at the time of spine surgery, as these drugs are known to affect neuromuscular function. EMG = electromyography, MEP = motor-evoked potential, TOF = train-of-four.

The disposition of patients is shown in Figure 1. Of the 366 patients who consented to participate at enrollment, 18 patients were excluded, and the analysis population consisted of 348 patients and 373 cases. Of these cases, there were 221 cases of sugammadex administration and 152 cases of spontaneous recovery.

The background characteristics of patients are summarized in Table 1. Among 373 cases, most (57.9%) were men, the mean (SD) age was 64.8 (13.1) years at the time of surgery, and the mean (SD) BMI was 23.9 (4.0) kg/m^2^. The sugammadex group was older than the spontaneous recovery group (mean [SD] age 67.3 [11.8] years vs 61.2 [14.1] years, respectively). The respective median (range) propofol, remifentanil, and fentanyl dosages were 2.50 (0.70, 3.60) μg/mL, 0.212 (0.080, 0.500) μg/kg/min, and 5.00 (0.90, 14.00) μg/kg in the sugammadex group and 2.55 (1.30, 4.40) μg/mL, 0.239 (0.050, 0.630) μg/kg/min, and 5.73 (1.30, 14.90) μg/kg in the spontaneous recovery group, and adequate depth of anesthesia was achieved in 85.3% of cases (deep, 29.0%; appropriate, 56.3%). Mean (SD) rocuronium doses used during surgery were 0.63 (0.19) and 0.50 (0.21) mg/kg based on actual weight in the sugammadex and spontaneous recovery groups, respectively. A few cases needed additional doses of rocuronium in the sugammadex group (4/221; 1.8%) and the spontaneous recovery group (9/152; 5.9%). The proportions of patients with renal impairment and hepatic impairment were higher in the sugammadex group versus the spontaneous recovery group (31.7% vs 25.7% and 35.7% vs 21.1%, respectively).

**Table 1:** Background characteristics of patients.

**Item**	**Sugammadex administration** **(n = 221**)	**Spontaneously recovered** **(n = 152**)	**Total** **(N = 373**)
Sex, male	128 (57.9)	88 (57.9)	216 (57.9)
Mean age at surgery (SD)	67.3 (11.8)	61.2 (14.1)	64.8 (13.1)
<40 yrs	6 (2.7)	16 (10.5)	22 (5.9)
≥40 to <50 yrs	18 (8.1)	16 (10.5)	34 (9.1)
≥50 to <60 yrs	24 (10.9)	25 (16.4)	49 (13.1)
≥60 to <70 yrs	61 (27.6)	42 (27.6)	103 (27.6)
≥70 yrs	112 (50.7)	53 (34.9)	165 (44.2)
Mean BMI (SD), kg/m^2^	24.0 (4.3)	23.7 (3.5)	23.9 (4.0)
Mean blood pressure (SD), mm Hg	72.2 (11.0)	67.0 (11.0)	70.1 (11.3)
Mean heart rate (SD), beat/min	67.1 (13.1)	70.9 (15.1)	68.7 (14.1)
ASA physical status classification			
Class 1	18 (8.1)	21 (13.8)	39 (10.5)
Class 2	166 (75.1)	115 (75.7)	281 (75.3)
Class 3	36 (16.3)	16 (10.5)	52 (13.9)
Unknown	1 (0.5)	0 (0.0)	1 (0.3)
Surgical procedures			
Anterior fusion	8 (3.6)	12 (7.9)	20 (5.4)
Posterior fusion	74 (33.5)	39 (25.7)	113 (30.3)
Laminoplasty	75 (33.9)	43 (28.3)	118 (31.6)
Laminectomy	30 (13.6)	18 (11.8)	48 (12.9)
Tumor removal	11 (5.0)	19 (12.5)	30 (8.0)
Nucleotomy	14 (6.3)	3 (2.0)	17 (4.6)
Orthodontics	3 (1.4)	7 (4.6)	10 (2.7)
Marmot	2 (0.9)	6 (3.9)	8 (2.1)
Others*	3 (1.4)	5 (3.3)	8 (2.1)
Not recorded	1 (0.5)	0 (0.0)	1 (0.3)
Affected areas			
Cervical	60 (27.1)	58 (38.2)	118 (31.6)
Thoracic	26 (11.8)	18 (11.8)	44 (11.8)
Lumbar	113 (51.1)	47 (30.9)	160 (42.9)
Others (2 or more areas)	22 (10.0)	29 (19.1)	51 (13.7)
Preoperative motor paralysis			
No	101 (45.7)	60 (39.5)	161 (43.2)
Yes	102 (46.2)	82 (53.9)	184 (49.3)
Unknown	18 (8.1)	10 (6.6)	28 (7.5)
BIS	n = 152 43.9 (8.4)	n = 128 42.9 (10.7)	n = 280 43.5 (9.5)
PSI	n = 48 29.5 (8.9)	n = 5 42.4 (21.4)	n = 53 30.7 (11.0)
BIS/PSI			
Deep anesthesia	62 (28.1)	46 (30.3)	108 (29.0)
Appropriate anesthesia	132 (59.7)	78 (51.3)	210 (56.3)
Light anesthesia	6 (2.7)	9 (5.9)	15 (4.0)
Unknown	21 (9.5)	19 (12.5)	40 (10.7)
Mean core body temperature (SD), ˚C	36.0 (0.6)	36.0 (0.6)	36.0 (0.6)
Mean time from rocuronium administration to TOF ratio (left-APB) measurement (SD), min	51.6 (16.9)	47.3 (17.3)	49.7 (17.2)
Renal impairment	70 (31.7)	39 (25.7)	109 (29.2)
Chronic kidney disease stages†			
G1 (≥90)	33 (14.9)	35 (23.0)	68 (18.2)
G2 (≥60 to <90)	112 (50.7)	76 (50.0)	188 (50.4)
G3a (≥45 to <60)	48 (21.7)	22 (14.5)	70 (18.8)
G3b (≥30 to <45)	17 (7.7)	10 (6.6)	27 (7.2)
G4 (≥15 to <30)	4 (1.8)	1 (0.7)	5 (1.3)
G5 (<15)	7 (3.2)	8 (5.3)	15 (4.0)
Hepatic impairment	79 (35.7)	32 (21.1)	111 (29.8)
Diabetes	53 (24.0)	24 (15.8)	77 (20.6)
Cardiovascular disorders	26 (11.8)	21 (13.8)	47 (12.6)
Cerebrovascular disease	13 (5.9)	6 (3.9)	19 (5.1)
Use of aminoglycosides, yes	1 (0.5)	0 (0.0)	1 (0.3)
Use of calcium channel blockers, yes	75 (33.9)	49 (32.2)	124 (33.2)
Use of digitalis, yes	1 (0.5)	0 (0.0)	1 (0.3)
Use of antidepressant, yes	14 (6.3)	10 (6.6)	24 (6.4)
Use of diuretics, yes	10 (4.5)	5 (3.3)	15 (4.0)
TOF category‡			
<0.25	102 (46.2)	0 (0.0)	102 (27.3)
≥0.25 to <0.5	57 (25.8)	0 (0.0)	57 (15.3)
≥0.5 to <0.75	27 (12.2)	3 (2.0)	30 (8.0)
≥0.75 to <0.9	6 (2.7)	44 (28.9)	50 (13.4)
≥0.9	2 (0.9)	98 (64.5)	100 (26.8)
Unknown	27 (12.2)	7 (4.6)	34 (9.1)

Data are number (percentage) with the number of cases as the denominator unless otherwise stated. Other muscle relaxants, including succinylcholine, are not routinely used in Japan.

APB = abductor pollicis brevis, ASA = American Society of Anesthesiologists, BIS/PSI = bispectral index/patient state index, BMI = body mass index, eGFR = estimated glomerular filtration rate, SD, standard deviation, TOF, train-of-four.

* Other surgical procedures include nerve foramen, nail removal, left one side approach both sides decompression, spinal foreign body removal, and transforaminal lumbar interbody fusion.

† Chronic kidney disease (CKD) stages are based on eGFR (mL·min^ − 1^·1.73m^ − 2^): normal or high eGFR (G1), mild CKD (G2), moderate CKD (G3a), moderate CKD (G3b), severe CKD (G4), and end-stage CKD (G5).

‡ TOF category corresponds to measurements at the left-APB only

### 3.2. Primary analysis

The main patient background factors affecting the TOF ratio at the first MEP measurement after finishing the MEP setting (i.e., before sugammadex administration) in all patients (first primary objective) are shown in Table 2. Of the explanatory variables included in the final linear regression model, age (reduction of TOF ratio per year: −0.0062, 95% confidence interval [CI]: −0.0088, −0.0036; *P* < .0001), mean blood pressure (reduction by 1 mm Hg in blood pressure: −0.0056, 95% CI: −0.0087, −0.0025; *P* = .0004), hepatic impairment (reduction of TOF ratio compared with normal impairment: −0.0843, 95% CI: −0.1581, −0.0104; *P* = .0253), and rocuronium dose calculated for the patient’s ideal weight (reduction of TOF ratio per mg/kg: −0.8525, 95% CI: −1.0308, −0.6742; *P* < .0001) were identified as significant factors (*P* value <.05) affecting TOF ratio at the first MEP measurement after finishing the MEP setting.

**Table 2:** Primary analysis of factors affecting the TOF ratio (N = 334) and secondary analysis of factors affecting the MEP detection success rate (%) at the first MEP measurement after finishing the MEP setting (left-APB) (N = 359*): linear regression analysis and logistic regression analysis, respectively (Data from both sugammadex administration and spontaneously recovered groups were combined).

**Primary analysis** **Explanatory variables** †	**Item**	**Estimate**	**95% CI**		***P* value**
			Lower	Upper	
Age (yr)	Continuous	−0.0062	−0.0088	−0.0036	<.0001
Sex	0: Female/ 1: Male	-	-	-	-
BMI (kg/m^2^)	Continuous	0.0050	−0.0036	0.0136	.2538
Mean blood pressure (mm Hg)	Continuous	−0.0056	−0.0087	−0.0025	.0004
Heart rate (beat/min)	Continuous	0.0017	−0.0009	0.0042	.1962
Renal impairment	0: No/1: Yes	-	-	-	-
Hepatic impairment	0: No/1: Yes	−0.0843	−0.1581	−0.0104	.0253
Diabetes	0: No/1: Yes	-	-	-	-
Preoperative motor paralysis	0: No/1: Yes	0.0514	−0.0176	0.1204	.1442
Rocuronium dose (mg/kg) (ideal weight)	Continuous	−0.8525	−1.0308	−0.6742	<.0001
Propofol dose (μg/mL)	Continuous	-	-	-	-
Remifentanil dose (μg·kg^ − 1^·min^ − 1^)	Continuous	-	-	-	-
BIS^‡^/PSI§	1: Deep anesthesia (ref: appropriate)	-	-	-	-
	3: Light anesthesia (ref: appropriate)	-	-	-	-
Core body temperature (°C)	Continuous	−0.0351	−0.0940	0.0238	.2431
Time from rocuronium administration to TOF ratio (left-APB) measurement (min)	Continuous	0.0013	−0.0007	0.0032	.1931
**Secondary analysis** **explanatory variables** ∥	**Item**	**Odds ratio**	**95% CI**		***P*-value**
			**Lower**	**Upper**	
TOF ratio¶	<0.25 (ref: ≥0.9 category)	0.0922	0.0437	0.1944	<.0001
	≥0.25 to <0.5 (ref: ≥0.9 category)	0.3402	0.1698	0.6819	.0024
	≥0.5 to <0.75 (ref: ≥0.9 category)	0.1713	0.0595	0.4930	.0011
	≥0.75 to <0.9 (ref: ≥0.9 category)	1.3785	0.6708	2.8327	.3824
BIS^‡^/PSI§	Deep anesthesia (ref: appropriate)	1.3375	0.7744	2.3100	.2969
	Light anesthesia (ref: appropriate)	1.5626	0.4768	5.1214	.4611

Missing values are imputed using a multiple imputation method prior to regression analysis. However, the amounts of rocuronium, propofol, and remifentanil are imputed by the last observation carried forward method.

* Multiple imputation method was applied to missing TOF ratios in 25 cases.

† Only the explanatory variables included in the final model are displayed for the stepwise (backward) analysis.

‡ BIS cutoff values: Deep (<40), appropriate (≥40 to ≤ 60), and light anesthesia (≥61).

§ PSI cutoff values: Deep (<25), appropriate (≥25 to ≤ 50), and light anesthesia (≥51).

∥ Only the explanatory variables included in the final model are displayed for the stepwise (backward) analysis. Missing values are imputed using the multiple imputation method prior to regression analysis.

**Figure 2.**
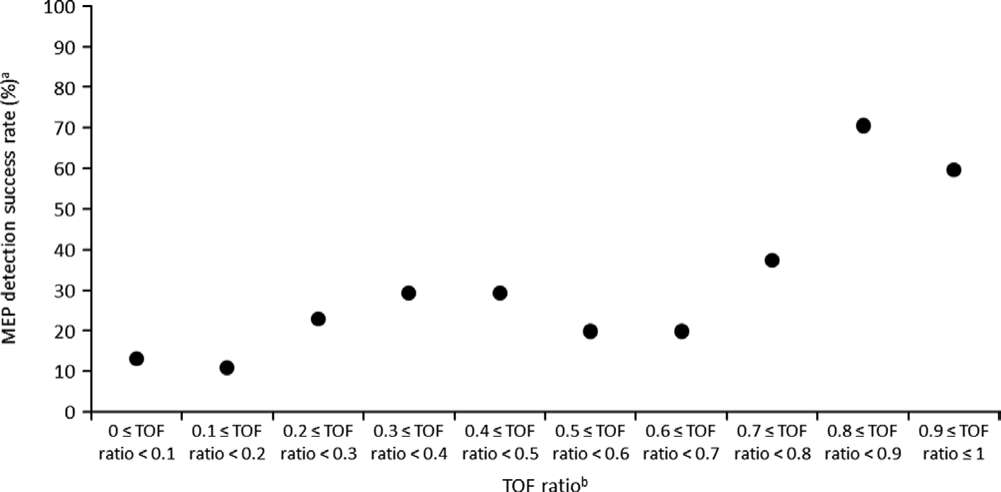
Relationship between TOF ratio and MEP detection success rate (N = 334). ^a^Overall MEP detection success rate at the first MEP measurement after finishing the MEP setting. ^b^The TOF was set in 0.1 increments to confirm the success rate of MEP detection for continuous changes in the TOF ratio. MEP = motor-evoked potential, TOF = train-of-four.

¶ The analysis results suggest that the cutoff value exists between 0.6 and 0.8, and 0.75 was indicated in Figure 2 and adopted as the threshold value.

APB = abductor pollicis brevis, BIS/PSI = bispectral index/patient state index, BMI = body mass index, CI = confidence interval, MEP = motor-evoked potential, TOF = train-of-four.

### 3.3. Secondary analysis

Figure 2 shows the relationship between the TOF ratio (left-abductor pollicis brevis) and MEP detection success rate at the first MEP measurement after finishing the MEP setting (second primary objective). The cutoff TOF ratio was 0.75 and patients with higher TOF ratios had higher MEP success rates. Of note, neither deep nor light anesthesia was found to affect the MEP detection success rate significantly.

In the post hoc regression analysis using a TOF ratio of ≥ 0.9 as the reference value, TOF ratios of <0.25 (odds ratio [OR]: 0.0922, 95% CI: 0.0437, 0.1944; *P* < .0001), ≥0.25 to <0.5 (OR: 0.3402, 95% CI: 0.1698, 0.6819; *P* = .0024), and ≥0.5 to <0.75 (OR: 0.1713, 95% CI: 0.0595, 0.4930; *P* = .0011) were identified as significant TOF ratio categories that affected the MEP detection success rate at the first MEP measurement after finishing the MEP setting. Meanwhile, the TOF ratio category ≥0.75 to <0.9 was not identified as statistically significant (*P* = .3824) (Table 2).

### 3.4. Other exploratory analyses

The TOF ratio was 0.284 immediately before the administration of sugammadex and then 0.929 15 minutes after administration. The time course profiles of the measured MEP amplitude and the amplification rate of the MEP amplitude are shown in Supplemental Digital Content 2, http://links.lww.com/MD/H440 and 3, http://links.lww.com/MD/H441, respectively. In patients treated with sugammadex, the MEP amplitude rapidly increased soon after sugammadex administration in all TOF ratio categories.

The factors affecting time-related variables, including time from intubation to start of MEP monitoring after NMB recovery, operation time, and operating room stay time, are shown in Table 3 and Supplemental Digital Content 4, http://links.lww.com/MD/H442 and 5, http://links.lww.com/MD/H443, respectively. The use of sugammadex was significantly associated with the shortening of all 3 time-related variables (all *P* < .0001). The least squares mean time from intubation to the start of MEP monitoring after NMB recovery was 62.34 minutes (95% CI: 53.63, 71.05) in the sugammadex group and 107.32 minutes (95% CI: 98.44, 116.21) in the spontaneously recovered group. The least squares mean difference between the sugammadex group and the spontaneously recovered group was − 46.07 minutes (95% CI: −60.06, −32.07; *P* < .0001).

**Table 3:** Factors affecting time between intubation and start of MEP monitoring measurement (minutes) (linear regression with the response variable set as the time from intubation to start of MEP monitoring) (N = 222).

**Explanatory variables** *	**Item**	**Estimate**	***P* value**
Sugammadex used	0: No/ 1: Yes	−46.0698	<.0001
Age (yr)	Continuous	0.4920	.0627
Rocuronium dose (mg/kg)	Continuous	30.3587	.1084
Propofol dose (μg/mL)	Continuous	11.9644	.0792
Time from rocuronium administration to TOF ratio (left-APB) measurement (minute)	Continuous	0.5698	.0022

APB = abductor pollicis brevis, MEP = motor-evoked potential, TOF = train-of-four.

* Only the explanatory variables with a *P*-value <.1 and rocuronium dose were included in the final model using a stepwise (backward) procedure.

This exploratory analysis also showed that the time from rocuronium administration to TOF ratio measurement was significantly associated with the time from intubation to start of MEP monitoring after NMB recovery and operating room stay time (*P* = .0022 and *P* = .0249, respectively) (Table 3 and Supplemental Digital Content 5, http://links.lww.com/MD/H443). Furthermore, BMI and propofol dose were found to be significantly associated with operation time (*P* = .0399 and *P* = .0042, respectively) and operating room stay time (*P* = .0374 and *P* = .0085, respectively) (Supplemental Digital Content 4, http://links.lww.com/MD/H442 and 5, http://links.lww.com/MD/H443).

## 4. Discussion

Although the usefulness of sugammadex in patients with prolonged muscle relaxants at the first MEP measurement after finishing the MEP setting has been previously studied,^[10,15]^ this study is novel in that it analyzed a relatively large sample of only Japanese patients under real-world settings (over 300 patients). Furthermore, the results of the multivariable analyses can be generalized to some extent, as the analysis was conducted on a relatively large population. Among the time-related measures, the results of sugammadex and spontaneous recovery groups were comparable, even though the proportion of patients with factors that prolong the action of rocuronium was higher in the sugammadex group versus the spontaneous recovery group (i.e., elderly people and those with renal or hepatic dysfunction), thus validating the effectiveness of sugammadex.

In the present study, the TOF ratio was affected by patient age, mean blood pressure, hepatic function, and rocuronium dose. Based on the metabolism of rocuronium (i.e., it requires hepatic sequestration for 80% of its elimination^[16]^), it is not surprising that hepatic function would affect the TOF ratio during NMB with rocuronium. A recent study compared the effects of age on rocuronium kinetic disposition in American Society of Anesthesiologists Class 1 to 3 patients undergoing elective surgeries and found that elderly patients had increased area under the curve/dose and reduced total clearance compared with young adults.^[17]^ Although blood pressure was also identified as a significant factor, this finding may have low clinical significance. Patients with high blood pressure had low TOF; this finding could be attributed to the effects of hypertension medication such as calcium channel blockers, which may enhance the effect of rocuronium. In this study, about 33% of patients were taking calcium channel blockers on the day of surgery; however, this speculation requires further confirmation.

In this study, a higher TOF ratio indicated a higher MEP detection success rate, and the TOF cutoff for the MEP detection success rate was 0.75 (TOF ratio ≥0.75). MEPs can be detected adequately at a TOF ratio of ≥0.75 for the abductor pollicis brevis, tibialis anterior, soleus, and abductor hallucis muscles, which is the basis for the TOF ratio of 0.75 used in previous studies.^[5,18–20]^

When evaluating the factors affecting time-related variables (time from intubation to start of MEP monitoring after NMB recovery, operation time, and operating room stay), the use of sugammadex was found to be significantly associated with all 3 time-related variables (all *P* < .0001) even though our study set a conservative 15 minutes after sugammadex administration to begin MEP monitoring for surgery. The regression analysis showed that the spontaneous recovery group had a longer waiting time for surgery monitoring than the sugammadex group. Although the cause of shortened operation time may be related to several factors and the extent of the contribution of sugammadex is unknown, we consider that the main reason is the shortening of the time from intubation to the start of MEP monitoring after recovery. It is also possible that because the MEP setting is finished and the MEP monitoring is started before surgery begins, less time is needed to monitor MEPs during surgery. Other reasons may be that drug failure or failure of the hardware needed to monitor MEPs can be readily detected before surgery, thus avoiding any delays during surgery. A meta-analysis study showed that sugammadex reduced the time in the operating room by several minutes compared with neostigmine when these agents were used to reverse NMB induced by rocuronium or vecuronium.^[21]^

Although several patients received additional rocuronium doses based on body movements, the proportion of patients requiring the additional dose was greater in the spontaneous recovery group. Given the small number of cases and the nature of this study, we did not investigate the safety or the impact of additional doses of rocuronium. However, the appropriate dose of sugammadex should be based on the TOF ratio. Here we show that the TOF ratio threshold for MEP detection success rate after finishing the MEP setting was ≥0.75. Furthermore, because we did not assess safety, this cutoff point of 0.75 should be considered in terms of a risk-benefit evaluation in the future.

The main limitations of this study were the observational design, the single-center study conduct, and inclusion of patients undergoing spine surgery only. Nevertheless, long-term continuous data were collected for a broad range of patients. Although the population was representative of patients treated at our center, the results may not be generalizable. Retrospective data collection of some parameters may not have been consistently captured, defined, or recorded. There may be selection bias as only data from patients who gave informed consent were included in the analysis. Adaptive confounding between the sugammadex dose and natural recovery makes it challenging to identify factors that influence the choice of sugammadex treatment during the assessment of exploratory objectives and outcomes. Considering these preoperative factors, administering an optimal amount of muscle relaxant and evaluating NMB status by monitoring parameters such as TOFs before starting MEP monitoring might improve the reliability of intraoperative MEP monitoring. While the surgeons and monitoring staff were proficient, the broad range of operative procedures performed could have impacted time-related data.

The present results suggest that age, blood pressure, hepatic impairment, and rocuronium dose were the main factors affecting the TOF ratio at the first MEP measurement after finishing the MEP setting. Administering an optimal amount of muscle relaxant considering these preoperative factors and evaluating the NMB state based on TOFs before starting MEP monitoring can improve the reliability of intraoperative MEP monitoring. TOF ratio categories (<0.25, 0.25 to <0.5, 0.5 to <0.75, 0.75 to <0.9, and ≥0.9) were related to MEP detection success. Patients with a higher TOF ratio (i.e., ≥0.75) had a high MEP detection success rate. The exploratory analyses indicated that MEP amplitude rapidly increased shortly after administering sugammadex in all TOF ratio categories in patients. Additionally, the time from intubation to the start of MEP monitoring after NMB recovery, operation time, and operating room stay time may be shortened by sugammadex. As adequate recovery from muscle relaxation is important, sugammadex administration before surgery may help improve the detection of MEPs during surgery.

## Acknowledgments

The authors wish to thank Keyra Martinez Dunn, MD, and Michelle Belanger, MD, of Edanz (www.edanz.com), for providing medical writing support, which was funded by MSD K.K., Tokyo, Japan.

## Author contributions

**Conceptualization:** Hironobu Hayashi, Miki Yamada, Kotoba Okuyama, Tsunenori Takatani, Hideki Shigematsu, Yasuhito Tanaka, Masahiko Kawaguchi.

**Data curation:** Hironobu Hayashi, Miki Yamada, Kotoba Okuyama, Masahiko Kawaguchi.

**Formal analysis:** Kotoba Okuyama.

**Writing – original draft:** Miki Yamada, Kotoba Okuyama, Masahiko Kawaguchi.

**Writing – review & editing:** Hironobu Hayashi, Miki Yamada, Kotoba Okuyama, Tsunenori Takatani, Hideki Shigematsu, Yasuhito Tanaka, Masahiko Kawaguchi.

## Supplementary Material


