# Supplementary figures and images for: Retrospective observational study of the effects of residual neuromuscular blockade and sugammadex on motor-evoked potential monitoring during spine surgery in Japan
Source: Medicine (Baltimore). 2022 Sep 30;101(39):e30841. doi: 10.1097/MD.0000000000030841 (PMC9524887; doi:10.1097/MD.0000000000030841)

Supplementary  
Digital Content 1.

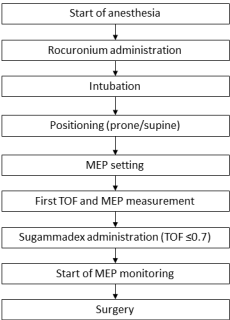

Supplement: Supplementary file 1 [file medi-101-e30841-s001.pdf]
