# Supplementary material for: Retrospective observational study of the effects of residual neuromuscular blockade and sugammadex on motor-evoked potential monitoring during spine surgery in Japan
Source: Medicine (Baltimore). 2022 Sep 30;101(39):e30841. doi: 10.1097/MD.0000000000030841 (PMC9524887; doi:10.1097/MD.0000000000030841)
Supplement: Supplementary file 2 [file medi-101-e30841-s002.pdf]

Supplementary Digital Content

Supplemental Digital Content 2. Table. Time course of the measured value of MEP amplitude: left-APB stratified by TOF ratio categories (sugammadex administration and spontaneously recovered)

		Sugammadex administration							Spontaneously recovered		
		**Baseline**	**5 minutes**	**10 minutes**	**15 minutes**	**30 minutes**	**60 minutes**	**120 minutes**	**Baseline MEP**	**MEP before surgery**	**Left-APB-TOF after surgery**
TOF ratio <0.4	N	74	13	37	52	68	66	39	0	0	0
	Mean	420.94	1322.69	1904.16	1965.90	1920.09	1916.71	1992.65	-	-	-
	Std	601.96	885.08	1963.81	1967.14	1934.11	1750.10	1666.35	-	-	-
	Median	165.00	1380.00	1300.00	1330.00	1245.00	1396.50	1500.00	-	-	-
	(Min, Max)	(0.0, 2930.0)	(0.0, 3380.0)	(0.0, 9120.0)	(0.0, 8340.0)	(0.0, 8058.0)	(0.0, 7340.0)	(0.0, 5300.0)	-	-	-
TOF ratio ≥0.4 to <0.6	N	19	6	6	15	19	14	13	0	0	0
	Mean	248.76	962.67	1546.83	1255.27	1400.58	1195.43	1363.92	-	-	-
	Std	350.07	1345.04	1523.57	1540.61	1349.91	1290.67	1438.25	-	-	-
	Median	210.00	354.50	1330.00	720.00	806.00	657.50	513.00	-	-	-
	(Min, Max)	(0.0, 1314.0)	(0.0, 3430.0)	(157.0, 3925.0)	(0.0, 5150.0)	(0.0, 4300.0)	(0.0, 3990.0)	(0.0, 4533.0)	-	-	-
TOF ratio ≥0.6 to <0.8	N	10	2	4	8	9	7	5	8	8	8
	Mean	720.50	745.50	2652.75	1500.13	1469.67	1596.29	482.34	532.53	599.53	750.91
	Std	1013.87	374.06	2061.65	1913.70	1433.63	1356.89	496.16	556.95	713.32	956.44
	Median	347.50	745.50	2080.00	910.00	900.00	1220.00	341.00	266.00	266.00	260.65
	(Min, Max)	(0.0, 3420.0)	(481.0, 1010.0)	(840.0, 5611.0)	(0.0, 5757.0)	(30.0, 4400.0)	(84.0, 4300.0)	(27.7, 1150.0)	(0.0, 1595.0)	(0.0, 2131.0)	(0.0, 2821.0)
TOF											

Pr Proprietary

ratio											
≥0.8	N	2	1	2	1	1	2	0	129	129	129
	Mean	1144.50	5440.00	2740.00	5300.00	4320.00	2655.00	-	1553.72	1422.69	1307.95
	Std	1209.86	-	3535.53	-	-	2708.22	-	1814.36	1741.01	1524.45
	Median	1144.50	5440.00	2740.00	5300.00	4320.00	2655.00	-	907.00	656.00	699.00
	(Min, Max)	(289.0, 2000.0)	(5440.0, 5440.0)	(240.0, 5240.0)	(5300.0, 5300.0)	(4320.0, 4320.0)	(740.0, 4570.0)	-	(0.0, 8603.0)	(0.0, 8603.0)	(0.0, 6961.0)

Abbreviations: APB, abductor pollicis brevis; MEP, motor-evoked potential; TOF, train-of-four.
