# Supplementary material for: Retrospective observational study of the effects of residual neuromuscular blockade and sugammadex on motor-evoked potential monitoring during spine surgery in Japan
Source: Medicine (Baltimore). 2022 Sep 30;101(39):e30841. doi: 10.1097/MD.0000000000030841 (PMC9524887; doi:10.1097/MD.0000000000030841)
Supplement: Supplementary file 3 [file medi-101-e30841-s003.pdf]

Supplementary Digital Content

Supplemental Digital Content 3. Table. Time course of amplification rate of MEP amplitude: left-APB stratified by TOF ratio categories (sugammadex administration and spontaneously recovered)

		Sugammadex administration							Spontaneously recovered		
		**Baseline**	**5 minutes**	**10 minutes**	**15 minutes**	**30 minutes**	**60 minutes**	**120 minutes**	**Baseline MEP**	**MEP before surgery**	**Left-APB-TOF after surgery**
TOF ratio <0.4	N	74	12	27	38	48	47	29	0	0	0
	Mean	-	351.39	1543.63	871.26	682.53	911.31	983.09	-	-	-
	Std	-	522.81	4064.63	1489.78	1323.65	1592.47	2028.68	-	-	-
	Median	-	160.60	372.22	313.69	292.67	320.45	240.00	-	-	-
	(Min, Max)	-	(−100.0, 1750.0)	(−25.8, 20600.0)	(−100.0, 6876.7)	(−100.0, 8287.1)	(−84.8, 8529.0)	(−73.6, 9386.1)	-	-	-
TOF ratio ≥0.4 to <0.6	N	19	3	4	9	11	9	8	0	0	0
	Mean	-	263.58	408.06	615.22	918.86	889.80	1061.34	-	-	-
	Std	-	213.68	668.05	1016.35	2121.03	1865.16	2079.46	-	-	-
	Median	-	161.04	119.38	280.63	227.25	166.67	105.52	-	-	-
	(Min, Max)	-	(120.5, 509.2)	(−10.4, 1403.8)	(−63.4, 3128.9)	(−23.3, 7249.4)	(−19.4, 5779.5)	(−38.0, 6068.7)	-	-	-
TOF ratio ≥0.6 to <0.8	N	10	2	4	8	8	7	4	8	7	7
	Mean	-	270.00	461.01	206.71	296.74	226.78	123.81	-	4.80	21.77
	Std	-	283.73	164.51	275.38	309.32	240.23	290.30	-	12.70	39.75
	Median	-	270.00	416.53	205.17	177.30	199.64	21.99	-	0.00	20.00
	(Min, Max)	-	(69.4, 470.6)	(318.7, 692.3)	(−100.0, 607.6)	(−17.9, 854.2)	(−97.5, 681.7)	(−98.5, 549.7)	-	(0.0, 33.6)	(−48.6, 76.9)
TOF											

Proprietary

ratio											
≥0.8	N	2	1	2	1	1	2	0	129	122	122
	Mean	-	172.00	72.52	165.00	116.00	142.28	-	-	1.16	8.45
	Std	-	-	126.54	-	-	19.49	-	-	48.15	87.53
	Median	-	172.00	72.52	165.00	116.00	142.28	-	-	0.00	−13.89
	(Min, Max)	-	(172.0, 172.0)	(−17.0, 162.0)	(165.0, 165.0)	(116.0, 116.0)	(128.5, 156.1)	-	-	(−100.0, 237.3)	(−100.0, 506.2)

Abbreviations: APB, abductor pollicis brevis; MEP, motor-evoked potential; TOF, train-of-four.
