# Supplementary material for: Retrospective observational study of the effects of residual neuromuscular blockade and sugammadex on motor-evoked potential monitoring during spine surgery in Japan
Source: Medicine (Baltimore). 2022 Sep 30;101(39):e30841. doi: 10.1097/MD.0000000000030841 (PMC9524887; doi:10.1097/MD.0000000000030841)
Supplement: Supplementary file 4 [file medi-101-e30841-s004.pdf]

Supplementary Digital Content

Supplemental Digital Content 4. Table. Factors affecting operation time (minutes) (linear regression with the response variable set as operation time)

Explanatory variables[a]	Item	Estimate	p-value
Sugammadex used	0: No / 1: Yes	−69.8259	<0.0001
BMI (kg/m^2)	Continuous	4.2455	0.0399
Rocuronium dose (mg/kg)	Continuous	-	-
Propofol dose (µg/mL)	Continuous	47.7373	0.0042
Time from rocuronium administration to TOF ratio (left-APB) measurement (minute)	Continuous	0.7892	0.0951

[a] Only the explanatory variables with a p-value <0.1 and rocronium dose were included in the final model using the stepwise (backward) procedure.

Abbreviations: APB, abductor pollicis brevis; TOF, train-of-four.

Pr Proprietary
