# Supplementary material for: Retrospective observational study of the effects of residual neuromuscular blockade and sugammadex on motor-evoked potential monitoring during spine surgery in Japan
Source: Medicine (Baltimore). 2022 Sep 30;101(39):e30841. doi: 10.1097/MD.0000000000030841 (PMC9524887; doi:10.1097/MD.0000000000030841)
Supplement: Supplementary file 5 [file medi-101-e30841-s005.pdf]

Supplementary Digital Content

Supplemental Digital Content 5. Table. Factors affecting operating room stay time (minutes) (linear regression with the response variable set as operating room stay time)

Explanatory variables[a]	Item	Estimate	p-value
Sugammadex used	0: No / 1: Yes	−74.4931	<0.0001
BMI (kg/m^2)	Continuous	4.5501	0.0374
Rocuronium dose (mg/kg)	Continuous	-	-
Propofol dose (µg/mL)	Continuous	46.3651	0.0085
BIS[b]/PSI[c]	Deep anesthesia (ref: appropriate)	-30.0277	0.0997
	Light anesthesia (ref: appropriate)	21.0600	0.5873
Time from rocuronium administration to TOF ratio (left-APB) measurement (minute)	Continuous	1.1252	0.0249

[a] Only the explanatory variables with a p-value <0.1 and rocuronium dose were included in the final model using the stepwise (backward) procedure.

[b] BIS cutoff values: Deep (<40), appropriate (≥40 to ≤60), and light anesthesia (≥61).

[c] PSI cutoff values: Deep (<25), appropriate (≥25 to ≤50), and light anesthesia (≥51).

Abbreviations: APB, abductor pollicis brevis; TOF, train-of-four.

Pr Proprietary
